# MiRNA Profile Associated with Replicative Senescence, Extended Cell Culture, and Ectopic Telomerase Expression in Human Foreskin Fibroblasts

**DOI:** 10.1371/journal.pone.0012519

**Published:** 2010-09-01

**Authors:** Laura N. Bonifacio, Michael B. Jarstfer

**Affiliations:** Division of Medicinal Chemistry and Natural Products, Eshelman School of Pharmacy, University of North Carolina at Chapel Hill, Chapel Hill, North Carolina, United States of America; New Mexico State University, United States of America

## Abstract

Senescence is a highly regulated process that limits cellular replication by enforcing a G1 arrest in response to various stimuli. Replicative senescence occurs in response to telomeric DNA erosion, and telomerase expression can offset replicative senescence leading to immortalization of many human cells. Limited data exists regarding changes of microRNA (miRNA) expression during senescence in human cells and no reports correlate telomerase expression with regulation of senescence-related miRNAs. We used miRNA microarrays to provide a detailed account of miRNA profiles for early passage and senescent human foreskin (BJ) fibroblasts as well as early and late passage immortalized fibroblasts (BJ-hTERT) that stably express the human telomerase reverse transcriptase subunit hTERT. Selected miRNAs that were differentially expressed in senescence were assayed for expression in quiescent cells to identify miRNAs that are specifically associated with senescence-associated growth arrest. From this group of senescence-associated miRNAs, we confirmed the ability of miR-143 to induce growth arrest after ectopic expression in young fibroblasts. Remarkably, miR-143 failed to induce growth arrest in BJ-hTERT cells. Importantly, the comparison of late passage immortalized fibroblasts to senescent wild type fibroblasts reveals that miR-146a, a miRNA with a validated role in regulating the senescence associated secretory pathway, is also regulated during extended cell culture independently of senescence. The discovery that miRNA expression is impacted by expression of ectopic hTERT as well as extended passaging in immortalized fibroblasts contributes to a comprehensive understanding of the connections between telomerase expression, senescence and processes of cellular aging.

## Introduction

Senescence is a cellular state characterized by loss of replicative potential and continued metabolic activity that appears to function as a tumor suppressor mechanism but also contributes to aging. Several diverse stimuli including DNA damage, oncogene expression, and telomere attrition can lead to senescence. Even though diverse stresses are capable of inducing senescence, p53, Rb, and more recently Skp2 have been identified as critical pathways common to initiation, execution and maintenance of senescence-associated growth arrest [1], [2], [3]. Highlighting the importance of p53 in senescence and the role of senescence as a barrier against tumorigenesis, restoration of p53 activity in p53-depleted tumors can cause activation of senescence and tumor regression [4]. The critical pathways of senescence are controlled by a complex network that regulates chromatin remodeling, proliferation arrest, cell remodeling, activation of the senescence associated secretory pathway, and inhibition of apoptosis [2]. While major effectors of these critical pathways have been identified, a complete understanding of this molecular network is still limited.

Accumulating evidence suggests a role for microRNAs (miRNA) in conveying senescence. MiRNAs are small, 19-23 nucleotide, non-coding RNAs that repress the expression of target genes by either preventing translation of the target mRNA or causing its degradation. Recent work by Maes et al [5] describes the miRNA profile of replicative senescence in comparison to premature senescence and serum-starved cells using WI-38 fibroblasts. In this study we present the miRNA profile of replicative senescence in human BJ fibroblasts, which in contrast to WI-38 fibroblasts express negligible amounts of p16 [6], and compare this to the miRNA expression profile of BJ fibroblasts immortalized by the stable transfection of the catalytic subunit of human telomerase (hTERT). When the miRNA profile of senescent BJ cells (p16 deficient) is compared to the profile in WI-38 cells (p16 positive), a p16-independent senescence association of several miRNAs appears. In addition, we demonstrate the specificity of several miRNAs in senescence-induced growth arrest in BJ cells by comparing their expression to that observed in late passage immortalized BJ cells and wild type (WT) contact-inhibited quiescent BJ cells. Importantly, the observation that several miRNAs are down-regulated over time in BJ-hTERT cells (in contrast to their up-regulation during senescence in WT cells) and one miRNA is up-regulated in late-passage BJ-hTERT cells (in contrast to down-regulation during senescence) suggests that TERT can affect regulation of senescence-associated miRNAs. Finally, despite an abundance of evidence linking miR-34a to senescence [7], we demonstrate that this miRNA is up-regulated similarly in both senescent and late-passage BJ-hTERT cells. This may imply that programmed changes in miRNA expression associated with aging independent of senescence can regulate miR-34a expression, at least in BJ fibroblasts.

## Results

### Characterization of senescence and extended-passage WT and immortalized BJ cells

**Figure 1:**
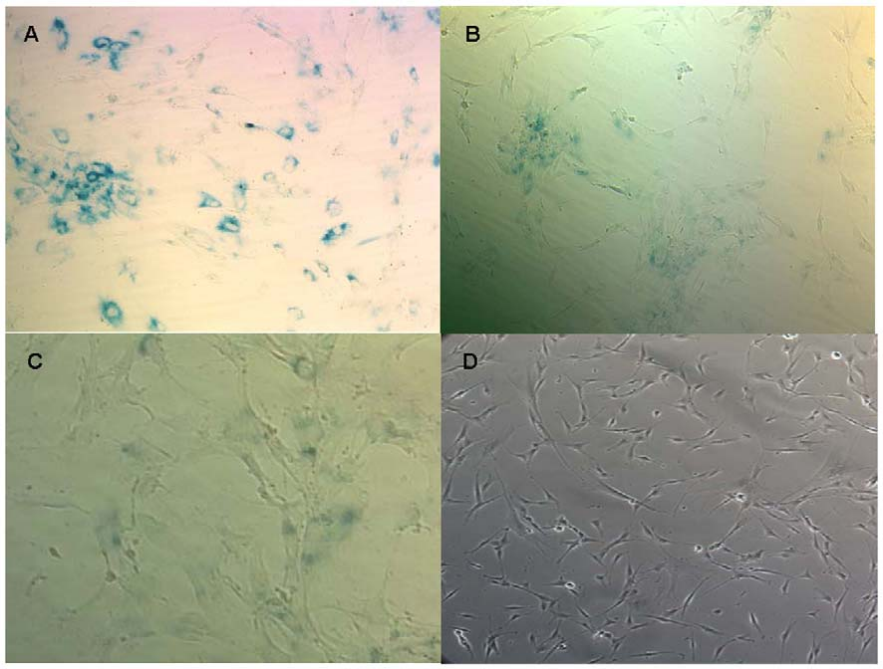
Beta-galactosidase staining in senescent WT and late passage immortalized cells. (A) Beta-galactosidase staining is shown as blue cells in senescent WT culture. (B) Staining is absent in the late-passage immortalized BJ cells. (C) Senescent BJ fibroblasts are flattened and enlarged compared to early passage WT BJs. (D) Late-passage BJ-hTERT cells do not display senescence-associated morphologic changes.

BJ fibroblasts were passaged to approximately 50 population doublings before population doubling time and morphologic changes indicated senescence in the WT cell line, and senescence was confirmed by beta-galactosidase staining (Fig. 1a, b). While the WT fibroblasts grew more slowly as they approached senescence, the immortalized BJ fibroblasts maintained a consistent population doubling time regardless of their passage age. Senescent wild type BJ cells were notably larger and flattened with increased lamellipodia compared to their early passage counterparts. The morphologic changes noted in the WT cell line during senescence were absent in the immortalized late-passage BJ cells (Fig. 1c, d).

### MiRNA profile of senescence in BJ fibroblasts

**Figure 2:**
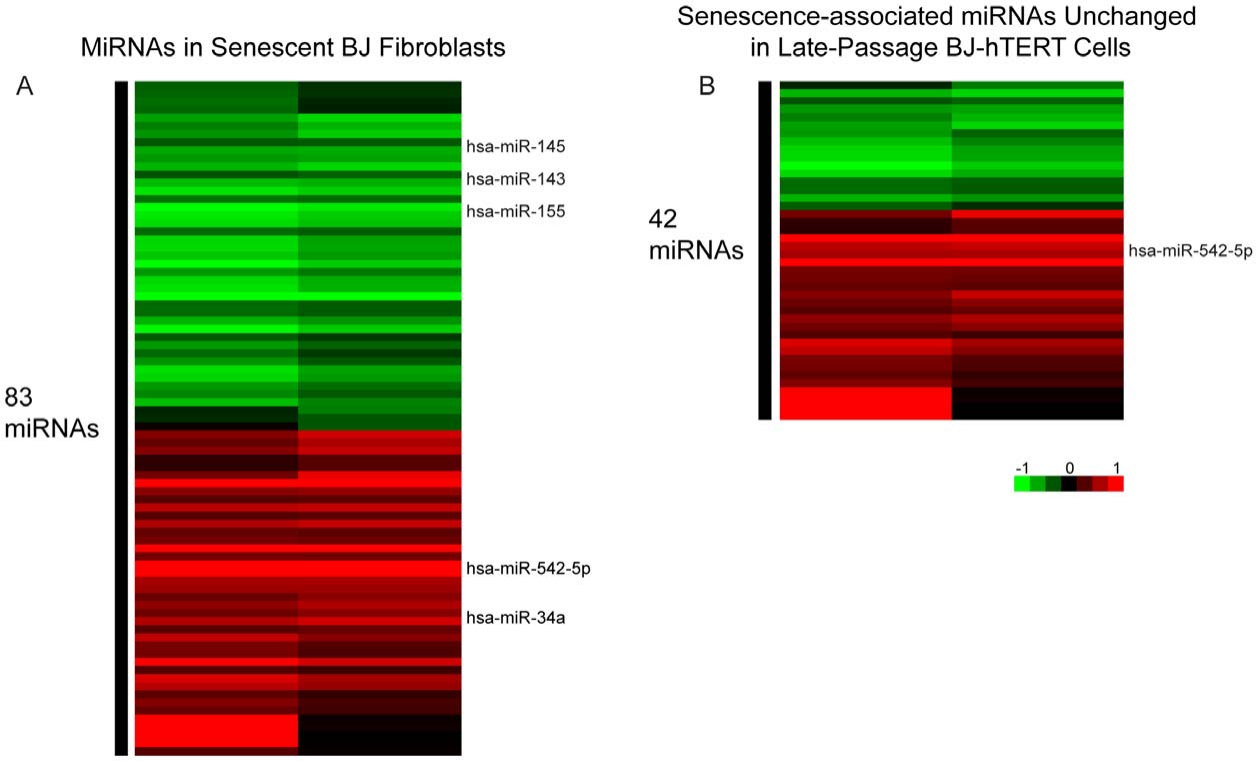
MiRNA expression in senescent BJ fibroblasts. Array results are depicted for each of the duplicate RNA samples used in the array. A) MiRNA microarray results reflecting those miRNAs whose expression in senescent cells differed by more than 1 standard deviation from the mean expression of each miRNA in early passage WT fibroblasts. B) Senescence-associated miRNAs that are not differentially expressed in late passage BJ-hTERT cells.

To identify those miRNAs that are differentially expressed during replicative senescence of BJ fibroblasts, we utilized a miRNA microarray platform that probes for expression of 470 human miRNAs and 64 human viral miRNAs, based on the Sanger miRNA database version 9.1. Microarray results reveal 83 miRNAs whose expression changed during senescence by more than 1 standard deviation compared to the mean expression of each miRNA in early passage WT fibroblasts (Figure 2a and Figure S1). Since each total RNA sample was arrayed in duplicate, one of the duplicate signals for a given miRNA must have indicated a change in expression of more than 1 standard deviation from the mean early passage signal for that miRNA to be identified as differentially expressed during senescence. To assist in parsing out those miRNAs that were changed during senescence due to a direct and specific senescence association, we compared and contrasted the array data from senescent BJ cells to BJ-hTERT cells that were passaged for an equal length of time (Figure 2b and Figure S2). Clearly, some miRNAs that were up-regulated in the senescent cells also were also up-regulated during extended culturing of the immortal cell line.

The microarray results corroborate suggested senescence-associated roles for several miRNAs. The miR-424-503 polycistron as well as miRs-450, 542-3p and 542-5p, which are all within 7 kb of the 424-503 polycistron, are significantly up-regulated in senescent BJ cells. This correlates well with previously published evidence indicating that miR-424 and 503 induce G1 arrest when overexpressed in human THP-1 cells by targeting several cell-cycle regulators [8]. Our data also reiterate the senescence-associated up-regulation of miR-373* and miR-663 and down-regulation of miR-197 observed in WI-38 cells [5] although less is known about the targets of these miRNAs.

We chose to validate the expression of several miRNAs during senescence with quantitative real-time PCR. The miRNAs chosen for validation were selected based on the significance of the microarray results and published evidence suggesting a role for the selected miRNAs in senescence. Our data confirm differential expression during senescence of several miRNAs for which considerable published evidence suggests a role in regulating senescence or proliferation-associated pathways, including miR-34a [7], [9], [10] and miR-146a [11], [12] (Table 1).

**Table 1:** Validation of select miRNA microarray results.

*MiRNA*	Senescent WT BJ fibroblasts*	Early passage BJ-hTERT cells*	Late passage BJ-hTERT cells*	Early passage quiescent WT BJ cells*
Let7c	0.3	0.8	0.7	0.7 ¥
10b	7.0 ¥	0.6	13.4 ¥	4.4 ¥
19a	1.5	3.3	3.5	1.5 ¥
21	0.4	1.9	2.3	0.7
23a	3.4 ¥	1.3	0.9	2.3 ¥
26a	2.9 ¥	1.1	1.2	2.1 ¥
34a	2.6 ¥	0.9	2.2 ¥	1.3 ¥
143	3.6	0.3	0.1	n.d.
145	3.4	0.3	0.1	1
146a	3.4 ¥	4.9 ¥	55.4 ¥	2.1
155	0.1	1	3.2	n.d.
199a-3p	3.2	1.4	1.7	2.2 ¥
542-5p	3.6 ¥	1.5	2.3	1.5

Quantitative real-time PCR was performed to validate selected microarray results. Late passage in the wild type cell line indicates senescent cells and results are shown relative to expression of U6.

*Values reflect expression relative to that in early passage WT BJ cells (set equal to 1).

¥ Denotes statistically significant values relative to 95% CI for experiments in early passage WT BJ cells. n.d. not detected.

We also validated the senescence-associated expression of several miRNAs with less abundant evidence for a senescence-associated function. We found that expression of miR-155, a proto-oncogenic miRNA [6], [8], is regulated during replicative senescence, consistent with the observed down-regulation in one study of aged WI-38 cells [13] and in aging in humans [14]. MiR-155 was ten-fold down-regulated during senescence of WT BJ fibroblasts. MiR-10b (a miRNA tied to invasion and metastasis in several cancer types) [15], [16], and miR-143 and miR-145 (polycistronic miRNAs that are down-regulated in tumors) [17] were also among the most significantly up-regulated (approximately 7-fold, 3.5-fold, and 3.5-fold, respectively) miRNAs during senescence.

### MiR-143 induces cell cycle arrest of WT BJ fibroblasts

**Figure 3:**
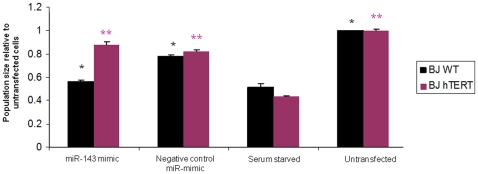
Effect of miR-143 on BJ fibroblast growth. Transient transfection of miR-143 mimic in WT BJ fibroblasts inhibits cell proliferation relative to untransfected cells at a level comparable to serum starved cells. MiR-143 does not inhibit proliferation of BJ-hTERT cells. One-way ANOVA indicates a lack of statistically significant variance between the BJ-hTERT samples denoted with purple double-asterisks. Similarly, a significant difference in variance exists between BJ WT samples denoted with black double-asterisks.

To determine whether miR-143 can affect the proliferation of BJ fibroblasts we transfected young BJ cells with a synthetic miR-143 mimic. The MiR-143 mimic inhibited the proliferation of young BJ cells in a dose-dependent manner. At a concentration of 60 nM, miR-143 inhibited proliferation to an extent that was statistically indistinguishable from growth inhibition caused by serum starvation (Figure 3). Analysis by one-way ANOVA comparing growth of miR-143 transfected cells to control and untransfected cells suggests that transfection with a miR-143 mimic results in a significantly smaller cell population 3 days after transfection. Remarkably, identical experiments in BJ-hTERT cells indicate that hTERT expression renders BJ cells resistant to miR-143 induced cell-cycle arrest (Figure 3). Preliminary evidence suggests this result is not cell-type specific, since another foreskin fibroblast cell line, NHF1, transfected with hTERT are also resistant to miR-143 (Bonifacio and Jarstfer, unpublished data).

### Expression of senescence-associated miRNAs during quiescence

In order to determine if the miRNAs validated as being differentially expressed during replicative senescence are associated specifically with replicative senescence pathways or more broadly with cell cycle arrest, we used real-time PCR to reveal expression levels of selected miRNAs in early passage, quiescent BJ fibroblasts. For this application, RNA was isolated from BJ WT cells that were population doubling seven and maintained in a confluent (contact inhibited) state for 3 days. Of the miRNAs we identified as being differentially regulated during senescence and validated with RT-PCR, 6 were confirmed to be either up-regulated in senescence whereas they remained unchanged or down-regulated, in the case of miR-143, in quiescent cells (Table 1). MiR-146a is up-regulated 3.4-fold during replicative senescence and lacks a significant change in expression during quiescence. MiR-145, which is approximately 3.5-fold up-regulated during senescence, is undetected in the quiescent samples. The expression of its polycistronic counterpart, miR-143, is unchanged during quiescence relative to expression in early passage cells. MiR-23a, a miRNA capable of inducing apoptosis in HEK cells [18], is 3.4 fold up-regulated during senescence and shows no change during quiescence. Three of the miRNAs, miR-199a-3p, miR-26a, and miR-10b, that we screened were up-regulated during both senescence and quiescence, and one, miR-155, was down-regulated in both senescent and quiescent samples

### MiRNA profile of extended passage immortalized BJ fibroblasts

**Figure 4:**
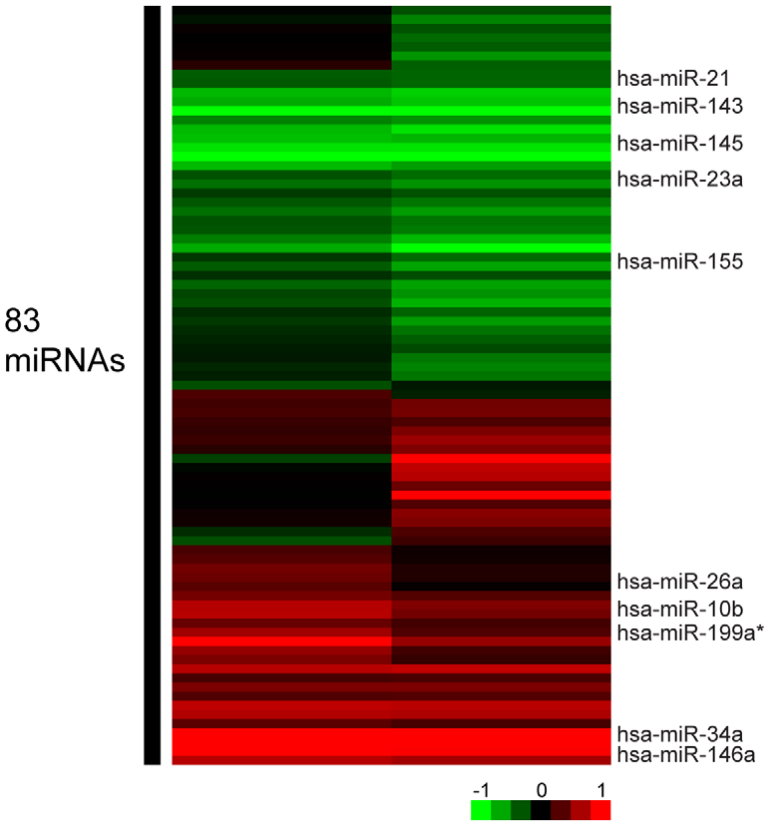
MiRNAs up-regulated during extended cell culture in BJ-hTERT cells. MiRNAs that are significantly up or down-regulated during extended passaging of BJ-hTERT cells. Array results are depicted for each duplicate of the late passage BJ-hTERT RNA sample.

To determine if changes in the miRNA footprint of senescent cells were related to extended cell culturing, we utilized a BJ cell line (BJ-hTERT) rendered immortal by the stable ectopic expression of hTERT, the telomerase catalytic subunit. BJ-hTERT cells experienced the same cell culture conditions as the WT cells and were only differentiated from the WT cell line by the expression of hTERT. RNA isolated from BJ-hTERT cells was used to reveal effects of long term cell culture in the presence of telomerase on miRNA expression. The expression of a few miRNAs increased significantly over time in the immortalized cell line in contrast to a small increase or a decrease over time in the wild type BJ cells (Figure 4 and Figure S3). One of the most significant examples of this is miR-155, an oncogenic miRNA [19]. MiR-155 was expressed at similar levels in early passage WT and immortalized BJ fibroblasts soon after transfection (Table 1). However, miR-155 levels increased approximately three-fold in the late passage BJ-hTERT cells whereas in senescent WT cells miR-155 decreased ten-fold relative to early passage WT cells. In addition, miR-146a increased 10-fold in the late passage immortalized cell line when compared to the early passage immortalized cells, whereas it increased only 3.4-fold in senescent WT cells. MiR-146a levels were also higher in early passage BJ-hTERT cells when compared to the early passage WT cells. Finally, whereas miR-143 and miR-145 were significantly up-regulated in senescent BJ cells, these miRNAs were down-regulated approximately 2 and 3-fold respectively in late-passage BJ-hTERT cells.

## Discussion

Senescence is the result of complex input from several pathways. Recent direct and indirect data indicate a role for miRNAs in regulating senescence [5], [11], [20], [21]. We have initiated an investigation of the roles for miRNAs in senescence by examining replicative senescence in BJ fibroblasts. Replicative senescence requires significant exposure to cell culture conditions, which potentially influences miRNA expression independently of senescence. For example, recent work reveals an age-related increase in DNA methylation in multiple cell types, including telomerase positive stem cells [22], [23]. Further, this hyper-methylation was shown to be present in both primary cell lines and extended-passage cell culture [24]. This data suggests the potential for programmed genetic changes that contribute to cellular aging independent from senescence. To differentiate the impact of extended culture on the miRNA profile from changes in miRNA expression related specifically to senescence, we compared the miRNAs of late passage immortalized BJ-hTERT cells to senescent WT BJ fibroblasts.

### MiRNAs with a Significant Link to Senescence Pathways

Of the 470 human miRNAs and 64 human viral miRNAs we screened, 83 showed differential expression in BJ fibroblasts during replicative senescence. Many of the miRNAs up-regulated in senescent BJ cells, are linked to senescence in various publications. For example, the miR-424-503 polycistron, miR-542-5p and 3p, and miR-450, all of which are likely to be part of the same primary transcript [8], are up-regulated significantly in senescent BJ cells. These results are consistent with previous reports showing that miR-424 and miR-503 are capable of inducing G1 arrest in multiple cell types [8], [25]. Our data also corroborate the up-regulation of miR-373* and miR-663 and down-regulation of miR-197 observed in senescent and quiescent WI-38 cells [5]. While little is known about the pathways regulated by these miRNAs, the fact that these miRNAs are regulated in replicative-senescent BJ cells implies a p16-independent function since senescence in BJ cells is orchestrated by p53 and not p16 [6].

Notably, we identified a pair of miRNAs with previously characterized roles in cancer cells, but an unclear role in regulating proliferation of normal human cells. MiR-143 and miR-145, which are processed from the same primary transcript, are up-regulated approximately 3.5-fold during senescence in WT BJ cells and either show no change or are down-regulated in the quiescent BJ cells. Further, both miRNAs are significantly down-regulated (approximately 10-fold) in late-passage BJ-hTERT cells. Together with the reported down regulation of miR-143 and miR-145 in several cancer cells, this suggest that miR-143 and miR-145 have a general role in regulating cellular proliferation and may function as tumor suppressor miRNAs. Consistent with this hypothesis, forced expression of these two miRNAs in cancer cells resulted in decreased growth [26]. We confirmed the ability of miR-143 to induce growth arrest in normal cells by transient over-expression of a miR-143 mimic in early passage BJ cells (Figure 3). Remarkably, miR-143 failed to arrest BJ-hTERT cells (Figure 3) and NHF1-hTERT cells (Bonifacio and Jarstfer, unpublished data). Perhaps expression of TERT results in up-regulation of one or more miR-143 targets, altering the concentration of miR-143 required to induce growth arrest. Alternatively, TERT has been implicated previously in pro-proliferative pathways separate from telomerase action at the telomere [27]. For example, TERT facilitates Wnt signaling via an interaction with BRG1 [28]. The influence of TERT in pro-proliferative pathways may obscure the anti-proliferative phenotype normally conferred by miR-143. The relationship between telomerase expression and inhibition of miR-143 induced growth arrest in fibroblasts is still being determined.

### MiRNAs that Increase During Extended Cell Culture Independently of Senescence

We made the surprising discovery that the expression pattern of some miRNAs changed differently overtime in late-passage immortalized BJ cells when compared to the WT senescent BJ cells. MiR-146a, which appears to function in a negative feedback loop to suppress the senescence associated secretory pathway [11], is expressed at 5-fold higher levels in early passage BJ-hTERT cells, relative to early passage WT cells, and undergoes an even more pronounced up-regulation in late passage BJ-hTERT cells (10-fold higher in late passage BJ-hTERT compared to senescent BJ cells). MiR-146a has been shown to down-regulate IRAK1 (part of the IL-1 signaling pathway) in response to inflammatory signaling that occurs during senescence [11]. Another major inflammatory signaler, the Wingless family (Wnt) proteins, participate in pathways controlling differentiation, inflammation, and tumorigenesis [29]. Evidence supports the altered regulation of Wnt genes in cells which have bypassed senescence and undergone transformation. For instance, the Wnt2B gene is in a chromosomal region known to be deleted and rearranged in a variety of cancers [30]. In addition, a recent report revealed that hTERT facilitates Wnt signaling by binding BRG1, a histone remodeling protein that signals through the β-catenin pathway, to affect proliferation and cell survival of progenitor cells [28]. Perhaps ectopic expression of TERT in a somatic cell line such as BJ fibroblasts stimulates robust Wnt and pro-inflammatory signaling resulting in up-regulation of miR-146a as part of a negative feed-back loop.

Another miRNA exhibiting a significant increase in expression over time in BJ-hTERT cells is miR-155. This is in contrast to a gradual decrease over time in WT BJ cells in which we observed an ultimate 10-fold down-regulation at senescence compared to expression during early passage populations. The down-regulation of miR-155 during senescence in BJ cells is consistent with published data indicating its role in promoting tumorigenesis [31]. MiR-155 expression is induced by a number of inflammatory mediators and is directly induced by AP-1 binding within its promoter [32]. AP-1 is a validated suppressor of the hTERT promoter in human cells [33]. It's possible that the over-expression of hTERT in BJ fibroblasts activates a negative feedback pathway that up-regulates AP-1, thereby inducing expression of miR-155. Alternatively, if ectopic expression of TERT engages the Wnt pathway as has been proposed [28], any number of the resultant up-regulated inflammatory modulators may be involved in the up-regulation of miR-155 in late-passage BJ-hTERT cells.

Although miR-34a has been linked to senescence via numerous publications [5], [34], [35], we demonstrate up-regulation of miR-34a in late-passage BJ-hTERT cells to a similar degree as that observed in senescent WT cells. Based on the previous observation that miR-34a is more frequently down-regulated in colorectal cancer cells compared to adenomas, in contrast to the frequent down-regulation of miR-143 and miR-145 in both cancer cells and adenomas, it has been implied that miR-143 and miR-145 regulate processes implicated in earlier phases of tumorigenesis [26]. Thus we postulate that miR-143 and miR-145 are critically involved in regulating the G1/S transition, whereas miR-34a may have broader roles in regulating stress response, including the stress of long-term cell culture conditions.

In conclusion, we profiled the miRNA expression in early passage and senescent human BJ fibroblasts and compared this profile to immortalized BJ cells that expresded the hTERT. The profiles identified the following subsets of miRNAs: miRNAs whose expression is regulated in the setting of replicative senescence in human foreskin fibroblasts devoid of substantial p16 activity and miRNAs whose expression is regulated over time in the presence of enforced hTERT expression. This accounting of miRNAs affected by the ectopic expression of TERT will expand the understanding of the relationship between telomerase, replicative senescence, and senescence-independent aging. We are currently investigating the roles of several of the identified miRNAs in controlling senescence-associated phenotypes and cellular proliferation.

## Materials and Methods

### Cell Culture

Human BJ foreskin fibroblasts (ATCC) were cultured at 37°C in a 5% CO_2_ incubator in MEMα supplemented with 1 mM sodium pyruvate, 1.5 g/L sodium bicarbonate, and 10% fetal bovine serum. Replicative senescence was induced by serial passage and was determined by observing an arrest in the growth rate, changes in cell morphology and senescence-associated β-galactosidase staining. HEK 293TS cells were used to generate retrovirus for stable transfection of hTERT.

### Immortalized BJ fibroblasts

BJ fibroblasts were immortalized by stable transfection with the catalytic subunit of human telomerase (hTERT). HEK 293T cells (a generous gift from Bryan Roth, UNC, originally from ATCC) were transfected with a pBabe-hygro vector modified to contain hTERT (a generous gift from Dr. Christopher Counter, Duke University) [36]. Twenty four hours after transfection, virus-laden media was used to infect BJ fibroblasts in the presence of 5 µl/ml polybrene and non-selective media. Twenty four hours after infection, the media was changed. Twenty four hours after the media change, the infected cells were split 1∶2 and transfected cells were selected with 100 µg/ml hygromycin. Expression of hTERT was verified in the immortalized cells by using the TRAPeze Telomerase Detection kit (Chemicon).

### Senescence-associated β-galactosidase staining

Cell staining was performed using a kit from Cell Signaling Technology with samples grown in a 6-well plate. After removing growth medium from the cells, the cells were washed with PBS and fixed with the manufactures fixative solution for 15 minutes at room temperature. The cells were then washed twice with PBS and stained with X-gal staining solution overnight at 37°C following the manufacturer's protocol.

### MiRNA microarray sample preparation, hybridization, and analysis

Total RNA was isolated from wild type early passage and senescent cells, as well as immortalized early and late passage cells using mirVana miRNA Isolation kit (Ambion). Sample quality was verified by measuring the ratio of 28S to 18S rRNA using the Agilent 2100 Bioanalyzer. 200 ng total RNA was dephosphorylated with CIP and labeled with pCp-Cy3. Labeled RNA was purified via spin column and hybridized to the Agilent miRNA microarray chip version 1. Detailed protocols can be found on www.agilent.com. Mean signal for each probe was quantile normalized and log_2_ transformed. Signals that mapped to the same miRNAs were collapsed into individual miRNAs by averaging. Each cell line high passage miRNA array was normalized to its corresponding low passage miRNA array. MiRNAs that differed one standard deviation or more from the their mean expression in early passage WT cells were identified. MiRNAs changing over time (specifically in the WT cell line and not the immortalized cells) were identified by selecting only those miRNAs that differed by more than one standard deviation in the WT BJs but not in the immortalized cells. The array data are MIAME compliant and have been deposited at the GEO database at the NCBI: accession number GSE22134.

### Quantitative real-time PCR

Selected miRNA microarray expression results were validated using the miRNA Taqman Assay (Applied Biosystems.) PCR experiments were performed in triplicate using the Applied Biosystems 7500 Real Time PCR instrument and normalized to the expression of U6. 10 ng of total RNA was reverse transcribed using a miRNA-specific primer then 1 µL of RT product was subjected to real-time PCR with a miRNA-specific probe.

### Transient transfection with miRNA mimics and SRB assay

We utilized the sulforhodamine B assay, described in detail in Kirtikara et al [37] to determine effects of miR-143 on growth of early passage BJ cells. BJ WT cells at population doubling number 5 were transfected with 60 nM miR-143 mimic or negative control miRNA mimic (Dharmacon) for 6 hours in Opti-MEMI in the presence of 3 µg/ml Lipofectamine 2000. After 6 hours, media was changed to normal BJ media without antibiotic. SRB assays were performed 72 hours after transfection. One way ANOVA was used to analyze the significance of variance between cell types from 3 separate experiments, each performed in quadruplicate.

## Supporting Information

Figure S1 MiRNA expression in senescent BJ fibroblasts. MiRNA microarray results reflecting those miRNAs whose expression differed by more than 1 standard deviation from the mean expression of each miRNA in early passage WT fibroblasts.
(6.53 MB TIF)

Figure S2 Senescence-associated miRNAs not affected by extended cell passaging. Some miRNAs changed in expression during senescence but not in late passage BJ-hTERT cells. Array results comparing senescent BJ fibroblasts to BJ-hTERT cells that were cultured for the same extended period are depicted for each of the duplicate RNA samples used in the array.
(4.65 MB TIF)

Figure S3 MiRNAs expression during extended cell culture in BJ-hTERT cells. MiRNAs that significantly change in expression during extended passaging of BJ-hTERT cells. Array results are depicted for each duplicate of the late passage BJ-hTERT RNA sample.
(7.98 MB TIF)
