# Supplementary figures and images for: MiRNA Profile Associated with Replicative Senescence, Extended Cell Culture, and Ectopic Telomerase Expression in Human Foreskin Fibroblasts
Source: PLoS One. 2010 Sep 1;5(9):e12519. doi: 10.1371/journal.pone.0012519 (PMC2931704; doi:10.1371/journal.pone.0012519)

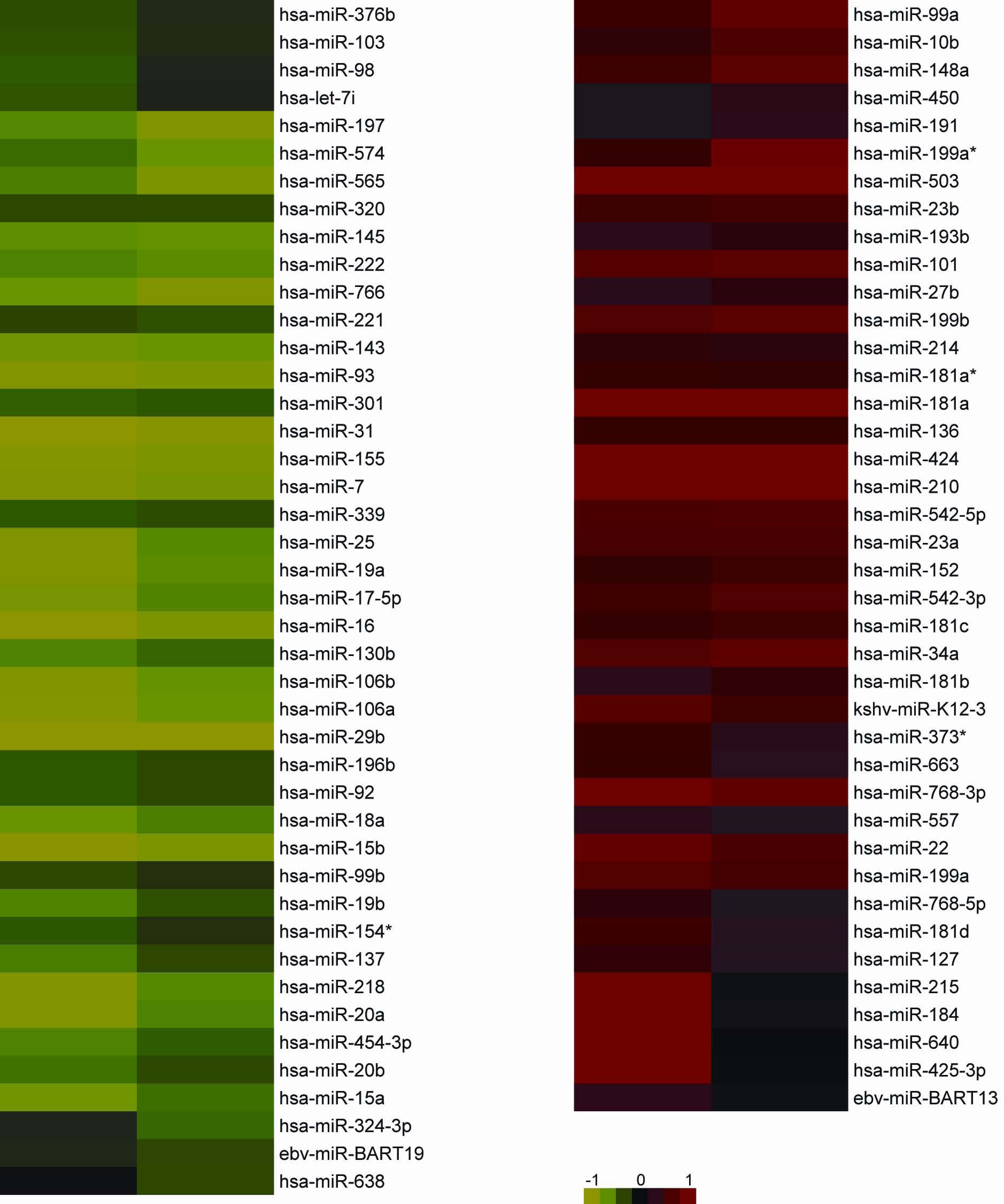

Supplement: Figure S1 — MiRNA expression in senescent BJ fibroblasts. MiRNA microarray results reflecting those miRNAs whose expression differed by more than 1 standard deviation from the mean expression of each miRNA in early passage WT fibroblasts. (6.53 MB TIF) [file pone.0012519.s001.tif]

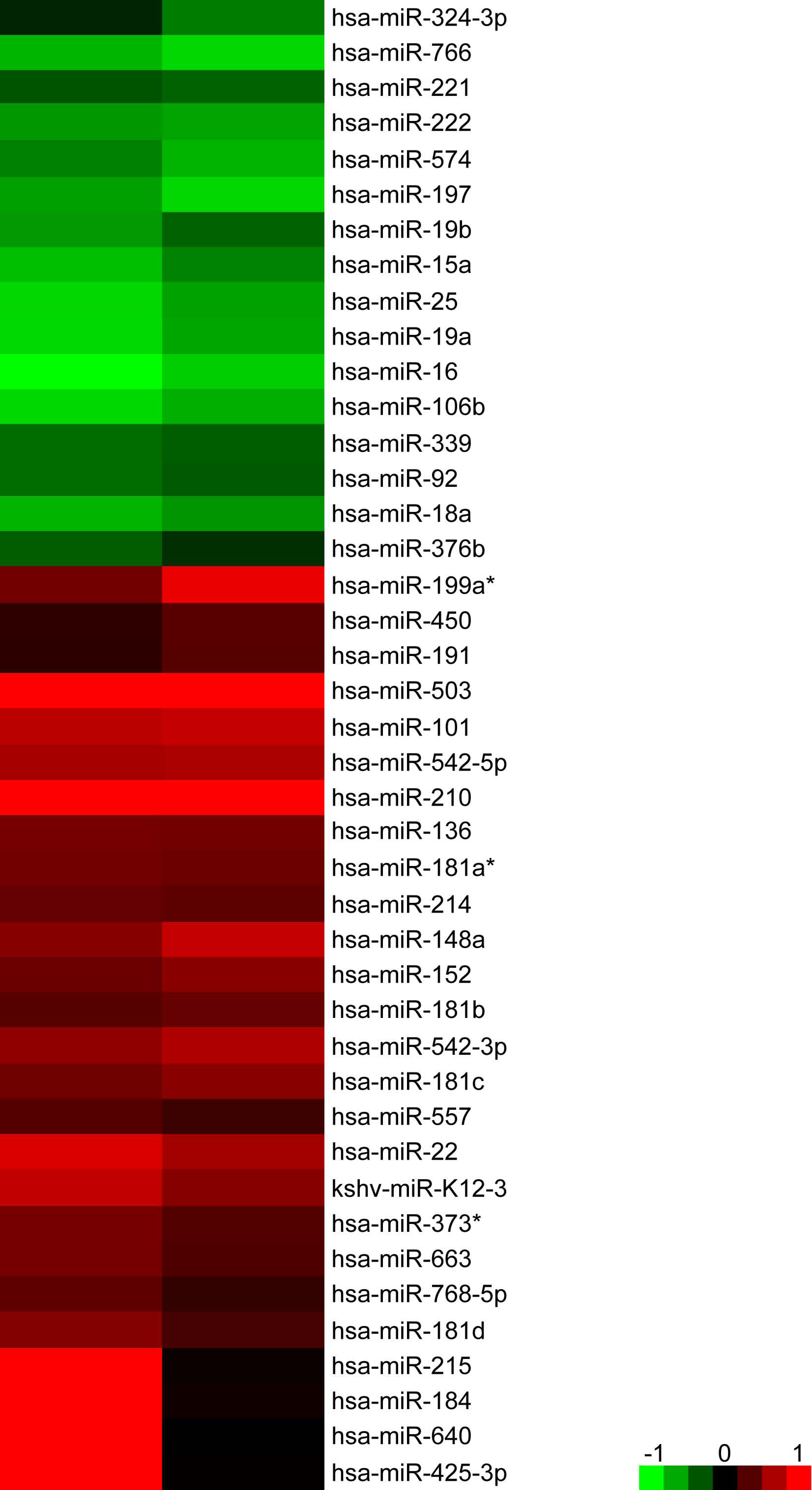

Supplement: Figure S2 — Senescence-associated miRNAs not affected by extended cell passaging. Some miRNAs changed in expression during senescence but not in late passage BJ-hTERT cells. Array results comparing senescent BJ fibroblasts to BJ-hTERT cells that were cultured for the same extended period are depicted for each of the duplicate RNA samples used in the array. (4.65 MB TIF) [file pone.0012519.s002.tif]

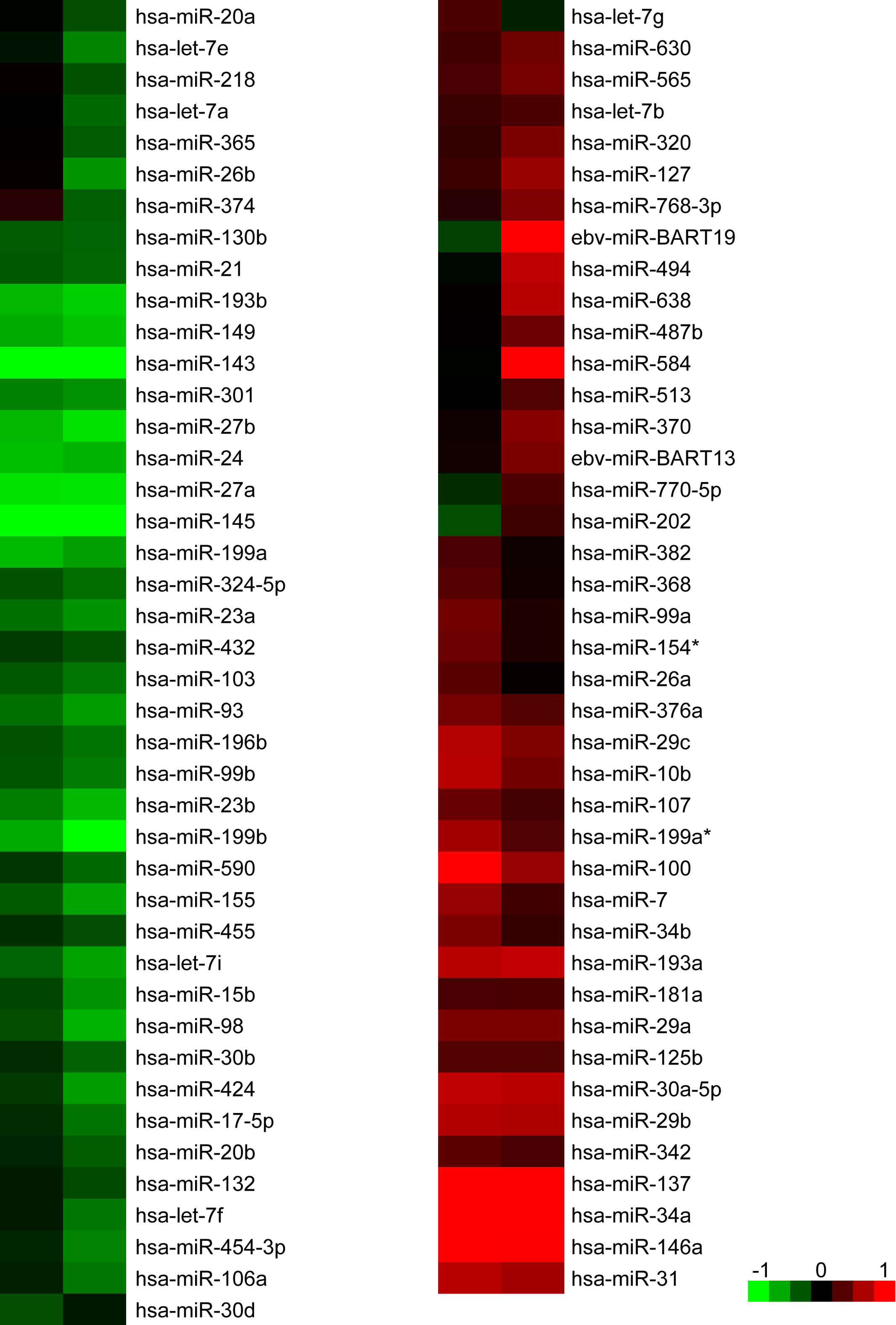

Supplement: Figure S3 — MiRNAs expression during extended cell culture in BJ-hTERT cells. MiRNAs that significantly change in expression during extended passaging of BJ-hTERT cells. Array results are depicted for each duplicate of the late passage BJ-hTERT RNA sample. (7.98 MB TIF) [file pone.0012519.s003.tif]
